# The role of sirtuin 1 and its activator, resveratrol in osteoarthritis

**DOI:** 10.1042/BSR20190189

**Published:** 2019-05-10

**Authors:** Zhenhan Deng, Yusheng Li, Haifeng Liu, Shengshi Xiao, Liangjun Li, Jian Tian, Chao Cheng, Greg Zhang, Fangjie Zhang

**Affiliations:** 1 Department of Orthopaedics, Xiangya Hospital, Central South University, Changsha, Hunan, China; 2 Department of Sports Medicine, The First Hospital Affiliated of Shenzhen University, Shenzhen Second People’s Hospital, Shenzhen, Guangdong, China; 3 Department of Emergency Medicine, Xiangya Hospital, Central South University, Changsha, Hunan, China; 4 National Clinical Research Center for Geriatric Disorders, Xiangya Hospital, Central South University, Changsha, Hunan, China; 5 Department of Joint Surgery, Zhuzhou Central Hospital, Zhuzhou, Hunan, China; 6 Department of Joint Surgery, Changsha Central Hospital, Changsha, Hunan, China; 7 Department of Orthopaedics, Yiyang Central Hospital, Clinical Medical Technology Demonstration Base for Minimally Invasive and Digital Orthopaedics in Hunan Province, Yiyang, Hunan, China; 8 McGovern Medical School, University of Texas Health Science Center at Houston, Houston, Texas, U.S.A.

**Keywords:** cartilage, chondrocyte, osteoarthitis, resveratrol, sirtuin 1

## Abstract

Osteoarthitis (OA) is the most common aging-related joint pathology; the aging process results in changes to joint tissues that ultimately contribute to the development of OA. Articular chondrocytes exhibit an aging-related decline in their proliferative and synthetic capacity. Sirtuin 1 (SIRT 1), a longevity gene related to many diseases associated with aging, is a nicotinamide adenine dinucleotide (NAD^+^)-dependent protein deacetylase and master metabolic regulator. Along with its natural activator resveratrol, SIRT 1 actively participates in the OA pathological progress. SIRT 1 expression in osteoarthritic cartilage decreases in the disease progression of OA; it appears to play a predominantly regulatory role in OA. SIRT 1 can regulate the expression of extracellular matrix (ECM)-related proteins; promote mesenchymal stem cell differentiation; play anti-catabolic, anti-inflammatory, anti-oxidative stress, and anti-apoptosis roles; participate in the autophagic process; and regulate bone homeostasis in OA. Resveratrol can activate SIRT 1 in order to inhibit OA disease progression. In the future, activating SIRT 1 via resveratrol with improved bioavailability may be an appropriate therapeutic approach for OA.

## Introduction

Osteoarthitis (OA), the most common aging-related joint pathology, is characterized by articular cartilage destruction along with changes occurring in other joint components, including bone, menisci, synovium, ligaments, capsule, and muscles [1]. In western populations, OA is one of the most frequent causes of pain, loss of function, and disability in adults [2]. The etiology of OA is mostly unclear, but several factors are suggested to be involved in the pathogenesis of OA, including mechanical, genetic, and aging-associated factors that ultimately lead to synovitis, apoptosis, and cartilage destruction. Advanced age is the greatest risk factor for OA [3]. Radiographic evidence of OA occurs in the majority of people by 65 years of age and in about 80% of those aged over 75 years [2]. The aging-related changes in joint tissues that contribute to the development of OA include cell senescence and aging changes in the extracellular matrix [4]. The sirtuins (SIRTs) family is a well-known group of antiaging genes [5]. It has been recently confirmed that the Silent information regulator 2 type 1 (also known as sirtuin 1 [SIRT 1]) is linked to various age-associated diseases such as obesity, type 2 diabetes, cardiovascular disease, cancer, dementia, arthritis, osteoporosis, as well as with OA [6]. It is essential to elucidate the roles of SIRT 1 and its natural activator, resveratrol, in the pathogenesis of OA in order to develop new successful approaches to the treatment of OA.

## Structure and basic function of SIRT 1

Nicotinamide adenine dinucleotide (NAD^+^) is a classical coenzyme mediating many redox reactions and an essential compound for many enzymatic processes [7]. In redox reactions, cellular levels of NAD^+^ are an important indicator of the cellular energy status; NAD^+^ can readily switch from the electron accepting form (oxidizing) NAD^+^ to the electron-donating form (reducing) NADH and vice versa [8]. SIRT 1 is an NAD^+^-dependent protein deacetylase and is a master metabolic regulator in different metabolic tissues [9].

The SIRTs are members of the silent information regulator 2 (SIR 2) family of highly conserved NAD^+^-dependent histone/protein deacetylases; they are a pivotal regulator of longevity and health span [10]. The SIRTs are associated with numerous cellular signaling pathways that include anti-inflammation, senescence, apoptosis, DNA damage repair, autophagy, and regulation metabolism in response to the cellular energy and redox status [11]. There are seven mammalian sirtuins, SIRT 1–7. SIRT 1 and SIRT 2 are localized in the nucleus and cytoplasm; SIRT 3, SIRT 4, and SIRT 5 are mitochondrial; and SIRT 6 and SIRT 7 are nuclear [12]. Each sirtuin contains a highly conserved catalytic core domain of approximately 275 amino acids which functions as a NAD^+^-dependent deacetylase and/or ADP-ribosyltransferase [13]. SIRT 1, the most-conserved mammalian NAD^+^-dependent protein deacetylase shares closest homology to yeast SIR 2. SIRT 1 splits NAD^+^ into nicotinamide and ADP-ribose, then transfers the acetyl group from the protein substrate to the 20-OH group of the ribose ring in the ADP-ribose molecule [9]. Histone deacetylases, in particular the sirtuin family with SIRT 1 as the major player, have long been linked to aging [14]. SIRT 1 is related to multiple age-associated diseases on account of its capacity to deacetylate histones and non-histone proteins such as tumor protein p53 (p53), kB-gene binding nuclear factor (NF-κB), heat shock factor 1 (HSF1), forkhead box transcription factor, class O (FOXOs), and peroxisome proliferator-activated receptor γ (PPARγ) coactivator-1 (PGC-1); thus, it’s able to regulate the cell’s biology, metabolism, and fate at different levels [15]. In mammalian cells, nutrient availability regulates the lifespan; p53, FOXO3a, and SIRT 1 – three proteins separately implicated in aging – constitute a nutrient-sensing pathway [16].

Resveratrol is a polyphenol found in the skin of red grapes and various other fruits, wines, peanuts, and root extracts of the weed Polygonum cuspidatum. It is thought to harbor major health benefits and is reported to be a substrate-specific activator of yeast SIR 2 and human SIRT 1 *in vivo* and *in vitro* [17]. Resveratrol is the most potent natural compound that activates SIRT 1, mimicking the positive effects of calorie restriction. In yeast, resveratrol mimics calorie restriction and increases DNA stability and extending lifespan by 70% [18]. In addition, resveratrol has shown to increase the lifespan of three model organisms through a SIR 2-dependent pathway [17,19]. Resveratrol increases cell survival by stimulating SIRT 1-dependent deacetylation of p53 [18]. Currently, aims to develop resveratrol with better bioavailability and targeting SIRT 1 at lower concentrations have shown promise [18].

## Expression of SIRT 1 in OA

The articular cartilage is an avascular, aneural, alymphatic, and viscoelastic connective tissue that derives its nutrition and oxygen supply by diffusion from the synovial fluid; along with subchondral bone, the articular cartilage is maintained at a low oxygen environment throughout life [20,21]. Chondrocytes are the only resident cells found in cartilage and are responsible for both the synthesis and turnover of the abundant extracellular matrix (ECM). Articular chondrocytes exhibit an age-related decline in their proliferative and synthetic capacity while maintaining the ability to produce pro-inflammatory mediators and matrices-degrading enzymes [22]. These findings are characteristic of the senescent secretory phenotype and are most likely a consequence of extrinsic stress-induced senescence driven by oxidative stress, rather than intrinsic replicative senescence. ECM changes, including the accumulation of proteins modified by non-enzymatic glycation, contribute to the propensity of developing OA [22,23].

Expression of the SIRT 1 protein is present in the nuclei of chondrocytes in all layers of the cartilage tissue as well as in synovial tissues [24,25]. All catabolic, mechanical, and nutritional stresses inhibit SIRT 1 expression [24]. Tumor necrosis factor-α (TNF-α), the main proinflammatory factor, could induce SIRT1 cleavage and reduce SIRT1 activity [26]. Oxidative stress-induced reduction of SIRT1 through post-translational modifications decrease SIRT1 activity and mark the protein for proteasomal degradation [27]. Accordingly, treatment with H_2_O_2_ results in the down-regulation of SIRT1 protein expression [28]. On the other hand, activation of the SIRT1 and related signaling pathway attenuates mitochondrial dysfunction and biogenesis [29], and defends against oxidative stress in articular chondrocytes [28].

It has been confirmed that SIRT 1 protein expression decreases in severely degenerated human cartilage, leading chondrocytes to hypertrophy and degeneration [30]. In patients with knee OA, expression levels of SIRT 1 are decreased in the articular cartilage (the lateral and medial sides of the tibia plateau including the loading zone and the margin zone) and is negatively associated with OA disease severity [30,31]. Moreover, SIRT 1’s downstream gene p53 expression and its acetylation level were dramatically increased in knee OA cartilage and is positively related to OA severity [31]. However, SIRT 1 expression was significantly reduced in human osteoarthritic subchondral osteoblasts compared with normal [32]. In contrast, SIRT 1 activity (cytoplasmic and nuclear) from peripheral blood mononuclear cells did not correlate with OA patients’ clinical activity (Lequesne’s index) or inflammation (erythrocyte sedimentation rate, C-reactive protein); in fact, it did not differ between patients with OA and healthy controls but instead correlates with the baseline interleukin (IL) -6 [33]. In wild-type mice with experimental knee OA, SIRT 1-positive chondrocytes are distributed from the superficial to the deep zone of the cartilage. Here, levels of SIRT 1 protein first increased but then gradually decreased with aging [34]. Synovial fluid from OA patients may contain proinflammatory cytokines including TNF-α, which could generate a stable and enzymatically inactive 75-kd form of SIRT 1. When human chondrocytes were exposed to OA-derived synovial fluid, the 75-kd SIRT 1 fragment was indeed generated, and levels of 75-kd SIRT 1 was elevated in OA versus normal chondrocytes [35].

## Effect of SIRT 1 in OA

### SIRT 1 regulates ECM

SIRT 1 seemsmicroM to play a predominant regulatory role in OA [36]. Expression of SIRT 1 in chondrocytes led to increased chondrocyte survival in either the presence or absence of TNF-α/actinomycin D [37]. Elevation of SIRT 1 protein levels or activity in human OA chondrocytes led to a dramatic increase in cartilage-specific gene (collagen II and aggrecan) expression; accordingly, 3D human chondrocytes present with both increased cellular SIRT1 enzymatic activity and COL2A1 expression [38,39]. Reduced expression of COL2A1 mRNA and type II collagen protein in human chondrocytes correlates with decreased SIRT 1 activity [39]. Another study confirmed SIRT 1 inhibition increases COL10A1 and ADAMTS5 (a disintegrin and metalloproteinase with thrombospondin motifs) expression while decreasing aggrecan expression [30]. It was discovered recently that glucosamine (GlcN) exhibits chondroprotective action on OA by enhancing the mRNA expression and protein levels of SIRT 1 and its downstream gene COL2A1 in chondrocytes [40].

### SIRT 1 promotes MSCs differentiation

SIRT 1 is required for promoting chondrogenic differentiation of mesenchymal stem cells (MSCs) [41]. It’s well known that sex determining region Y box protein 9 (SOX9) and runt-related transcription factor 2 (RUNX2) are the pivotal transcription factors in adult cartilage development [42]. SIRT 1 supports the chondrogenic development of MSCs at least in part through the inhibition/deacetylation of NF-κB and activation of SOX9 *in vitro* [41]. SIRT 1 may regulate the expression of RUNX2 and the production of matrix metalloproteinase (MMP) 13 from chondrocytes to adjust the hypertrophic chondrocyte lineage and degeneration of articular cartilage [43]. SIRT 1 deacetylates PPARγ and SOX9 to control the vav guanine nucleotide exchange factor 1 (Vav1), regulating MSC cell fate decisions for adipocyte and chondrocyte differentiation [44]. SIRT 1 is a major contributor of SOX9 deacetylation; the deacetylated state of SOX9 enables its importation to the nucleus and supports its transcriptional activity and transactivation of aggrecan [45]. SIRT 1 is active in the cartilage-specific transcription factor SOX9 and is dependent on NAD. Inhibition of nicotinamide phosphoribosyltransferase (NAMPT) leads to reductions in NAD levels, SIRT activity, and cartilage-specific gene expression. Therefore, SIRT 1, NAMPT, and NAD may provide a positive function in human cartilage by elevating the expression of genes encoding cartilage ECM [38]. SIRT 1 is also a key regulator of chondrocytes’ phenotype; IL-1β induces the de-differentiation of articular chondrocytes by the up-regulation of SIRT 1 activity enhanced by both NAMPT and extracellular signal-regulated kinases (ERK) signaling [46]. Decreased SIRT 1 in OA might lead chondrocytes to hypertrophy and degenerate [30]. SIRT1 plays an important role in MSCs’ differentiation and resistance to H_2_O_2_-induced oxidative stress during bone marrow-derived MSC (BM-MSC) osteogenesis [47,48]. In the SIRT1 RNAi cell model, knocking down the SIRT1 gene induced the Wnt signaling pathway, leading to the inhibition and decrease of cartilaginous proliferation and differentiation, but increasing apoptosis in ATDC5 cells [49]. Increased SIRT1 could inhibit adipogenesis and stimulate myogenic differentiation in MSCs through activating Wnt/β-catenin signaling [50,51]. Other factors were also involved in the process of SIRT1 regulation of MSC, such as the activation of the adenosine monophosphate-activated protein kinase (AMPK)-SIRT1 signaling pathway as well as beneficial mechanical stretch to induce antioxidant responses, attenuate intracellular reactive oxygen species (ROS), and improve osteogenesis of human BM-MSCs [52]. In mice, Sirt1 promotes MSC proliferation and osteogenic differentiation and inhibits MSC senescence via Bmi1 activation; therefore, treatment with resveratrol could promote bone formation and prevent bone loss [53]. SIRT1 was also directly involved in the regulation of beige adipocyte differentiation. Elevated SIRT1 prevents elderly adipose tissue-derived MSCs from entering senescence and restores the beige differentiation ability via the p53/p21 pathway [54].

### Anti-catabolic and anti-inflammatory effects

Previous studies confirmed that SIRT 1 exhibits anti-catabolic and anti-inflammatory effects in OA. Secreted inflammatory molecules, in particular the two major proinflammatory cytokines IL-1β and TNF-α, control the degeneration of articular cartilage matrix [55,56]. SIRT 1 and TNF-α appear to have opposing effects on cartilage gene expression; SIRT 1 expression or activity may be blocked in part by TNF-α [26]. TNF-α mediates the proteolytic cleavage of SIRT 1, producing a stable 75-kd SIRT 1 fragment that is incapable of binding chromatin and chromatin-associated coactivators, such as PGC-1 and SOX9 [26]. After the exposure of human chondrocytes to TNF-α, 75-kd SIRT 1 was exported to the cytoplasm and co-localized with the mitochondrial membrane, where the 75-kd SIRT 1 plays the role of preventing cell death through its enhanced association with cytochrome on the mitochondrial membrane to block downstream apoptosis by preventing apoptosome assembly and subsequent caspase 3 activation; 75-kd SIRT 1 is capable of promoting cell survival through an enzymatically independent mechanism [35]. Cartilage destruction in OA is thought to be mediated by two main enzyme families: the MMP enzymes are responsible for cartilage collagen breakdown, whereas the enzymes from the ADAMTS family mediate cartilage aggrecan loss [57]. Overexpression of SIRT 1 in human chondrocytes leads to the repression of MMP 3, -8, and -13 and ADAMTS 4 gene expression, and down-regulating SIRT 1 leads to the induction of MMP 13 [58]. In human chondrocytes treated with IL-1β, SIRT 1 can play a protective role by suppressing IL-1β-induced expression of cartilage-degrading enzymes such as ADAMTS 5, MMP 1, 2, 9, and 13 partially through the modulation of the NF-κB (p65) pathway [59]. When chondrocytes are incubated with TNF-α, SIRT 1 also activates, deacetylates, and inactivates NF-κB p65 to exert an inhibitive effect on the expression of cyclooxygenase-2 (COX-2), prostaglandin E2 (PGE2), and MMPs [60]. In human chondrocytes, fisetin inhibits IL-1β-induced expression of nitric oxide (NO), PGE2, TNF-α, IL-6, COX-2, inducible nitric oxide synthase (iNOS), MMP 3, MMP 13, ADAMTS 5, and remarkably suppressed the degradation of SOX9, aggrecan, and collagen-II; it exerts all these anti-inflammatory effects through activating SIRT 1 [61]. Silencing of microRNA-449a shows a protective effect via targeting SIRT 1 to inhibit catabolic gene expression, restoring anabolic gene expression in IL-1β-induced cartilage destruction [62].

### Anti-oxidative stress

SIRT 1 is strongly involved in the process of melatonin’s cytoprotective and anti-inflammatory effects in oxidative stress-stimulated chondrocytes. When oxidative stress induces senescence in chondrocytes, SIRT 1 enables chondrocytes to cope with unfavorable growing conditions. The mRNA of SIRT 1 was up-regulated after oxidant insult, but decreased in aging cells [63]. Expression of SIRT 1 could be induced by H_2_O_2_, and melatonin was confirmed to have the effect of decreasing SIRT 1 in chondrocytes [64]. Inhibiting SIRT 1 reversed the effects of melatonin on H_2_O_2_-mediated induction of proinflammatory cytokines (NO, PGE2, TNF-α, IL-1β, and IL-8) and the expression of iNOS and COX-2. Moreover, decreased SIRT 1 reversed the effects of melatonin, blocking the H_2_O_2_-induced phosphorylation of phosphoinositide 3-kinases (PI3K)/Akt, p38, ERK, C-Jun-N-terminal kinase (JNK), and mitogen-activated protein kinase (MAPK), as well as the activation of NF-κB [64]. In chondrocytes stimulated by oxidative stress, MiR-9 was identified and confirmed to be a post-transcriptional regulator of SIRT 1; MiR-9 silencing inhibits cell death, induced by H_2_O_2_ partly through down-regulation of SIRT 1 [65]. In H_2_O_2_-treated rat chondrocytes, rutin effectively inhibits the activation of inflammatory cytokines and MMP 2/9 by increasing SIRT 1, leading to the down-regulation of NF-kB/ MAPK, COX-2, and iNOS [28].

### Anti-apoptosis and participation in autophagy

Autophagy participates in the OA development and regulates changes in OA-like gene expression through modulation of apoptosis and ROS as a protective process [66]. SIRT 1 is also in involved in this progress. Hydroxytyrosol stimulates autophagy and offers protection from oxidative stress-induced cell death in a SIRT 1-dependent manner by increasing p62 transcription [67]. SIRT 1 is an anti-apoptotic protein in human chondrocytes on account of its enzymatic activity: expression of SIRT 1 leads to activation of the insulin-like growth factor (IGF) receptor (IGFR) and the downstream kinases PI3K, pyruvate dehydrogenase kinase 1 (PDK1), mammalian target of rapamycin (mTOR), and Akt, ultimately resulting in the phosphorylation of mouse double minute 2 homolog (MDM2), inhibition of p53, and blocking apoptosis [37]. Furthermore, in human chondrocytes, SIRT 1 regulates apoptosis through the modulation of mitochondria-related apoptotic signals via translocation of Bax and Bcl-2 (SIRT 1 inhibition increases the amount of Bax and reduces the amount of Bcl-2). However, the increased NO-induced apoptosis by SIRT 1 inhibition is mediated by the activation of caspases 3 and 9, but is independent of the caspase 8 pathway [24]. Both AMPK and SIRT 1 are strong inducers of autophagy. Meanwhile, homeostasis of mitochondrial mass through mitochondrial is maintained through biogenesis and mitophagy. In human OA chondrocytes, mitochondrial biogenesis is deficient, which is linked to reduced AMPK activity and decreased expression of SIRT 1. Activation of the AMPK/SIRT-1/PGC-1a pathway reversed the impaired mitochondrial biogenesis capacity in cultured human OA chondrocytes [68]. The SIRT 1/p53 signaling pathway showed direct involvement in the miR-34a regulation, apoptosis, and inhibition of cell proliferation in human chondrocytes [69]. In the process of ionizing radiation (IR) induction of cellular senescence of chondrocytes, the role that IR plays is negative post-translational regulation of SIRT 1 via ROS-dependent p38 kinase activation; up-regulation of SIRT 1 distinctly reduces the IR-induced senescence phenotype and vice versa [70].

### Other effects

In cartilage homeostasis, SIRT 1 also mediates the key clock gene expression with pathophysiological implications. In human knee OA cartilage, the levels of both NAD^+^ and Bmal 1, a circadian rhythm gene, were decreased significantly, resulting in the inhibition of NAMPT activity and SIRT 1 expression. Inhibition of SIRT 1 not only resulted in a reduction of Bmal1 and a moderate increase of period 2 (per2) and Rev-Erb α, but also further exacerbated the survival of cells with the expression of cartilage matrix-degrading enzymes induced by IL-1β [71].

**Figure 1:**
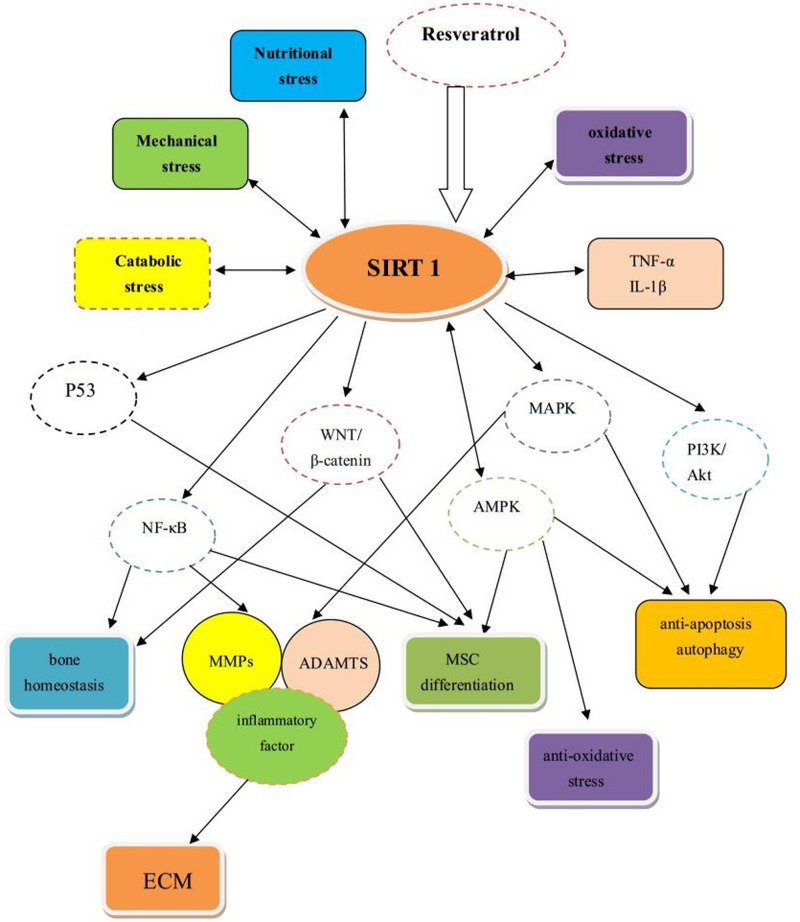
The mechanism of SIRT 1 and related pathway in OA → means there is a direct effect on the other, ↔ means there is an interaction between the both sides, ⇒ means there is an active effect on the other.

OA affects all joint components, not only the cartilage, but also the bone, synovium, and so on. SIRT 1 also plays an important role in bone homeostasis. SIRT 1 is a genetic determinant of bone mass: the lack of SIRT 1 promotes osteoclastogenesis in osteoclasts *in vitro* and reduces osteoblast differentiation in osteoblasts through the control of NF-κB and bone cell differentiation [72]. Decreased SIRT 1 levels were found in human osteoarthritic subchondral osteoblasts [32]. In addition, Calcar SIRT 1 expression in the osteoporotic femoral neck (calcar region) was significantly reduced while sclerostin was markedly increased, showing that SIRT 1 and sclersotin expression are inversely correlated [73]. Inhibition of SIRT 1 in osteoblasts leads to increased transforming growth factor-β1 (TGF-β1) and sclerostin expression that decreases Wnt/β-catenin activity; conversely, the stimulation of SIRT 1 reduces the expression of TGF-β1 and sclerostin, as well as increases the mineralization in OA osteoblasts [73]. Wnt/β-catenin signaling is important for normal bone homeostasis and function; osteoblasts and osteoclasts are affected by decreased sclerostin, the inhibitor of the Wnt/β-catenin signaling, and a SIRT 1 target [32]. The expression and production of SIRT 1 were decreased in OA subchondral bone tissue [74]. SIRT 1 may regulate apoptosis and ECM degradation via the Wnt/β-catenin signaling pathway in OA chondrocytes [75]. SIRT 1 can regulate the bone marrow adipocyte phenotype, inducing a thermogenic gene program in mouse and human BM-MSCs via sclerostin inhibition [76]. Due to the relationship between SIRT 1 and Wnt/β-catenin signaling, the disruptor of telomeric silencing 1-like (DOT1L) could directly control Wnt signaling by inhibiting the activity of SIRT1, playing the role of safeguarding the homeostasis in cartilage and protecting against OA [77]. In the process of deletion of the oxygen sensor prolyl hydroxylase (PHD) 2 in osteocytes, the enhanced SIRT1 activates the WNT/β-catenin signaling and decreases the sclerostin, leading to increased osteoblast number and activity while decreasing osteoclastogenesis and bone resorption. However, the expression and effects of SIRT 1 in osteoarthritic subchondral bone and synovium needs to be further investigated, the related mechanism of SIRT 1 in OA was shown in Figure 1.

### SIRT 1 in OA animal models

SIRT 1 has shown the ability to regulate the osteogenesis and adipogenesis of MSCs. MSC specific SIRT 1 knock-out (MSCKO) mice confirms that SIRT 1 regulates differentiation of MSCs by deacetylating β-catenin: MSCs isolated from MSCKO mice show reduced differentiation towards osteoblasts and chondrocytes *in vitro* [79]. In parathyroid hormone-related protein 1–84 [PTHrP(1–84)] knockin mice, Bmi-1 alters the BM-MSCs fate by enhancing osteoblast differentiation and inhibiting adipocyte differentiation, at least in part by stimulating SIRT 1 expression [80].

SIRT 1 and its enzymatic activity play a protective role in normal development and homeostasis of cartilage *in vivo* [81]. In the haploinsufficient SIRT 1 total body knockout (KO) mice, SIRT 1 KO mice exhibit cartilage defects that are consistent with their reduced size. SIRT 1 KO mice cartilage exhibit low levels of type II collagen, aggrecan, and glycosaminoglycan content in their paws; however, they exhibit elevated levels of MMP 13 and protein tyrosine phosphatase (PTP1B) in cartilage compamicroMred with wild-type (WT) mice [82]. Nevertheless, in the homozygous SIRT-1tm2.1Mcby (SIRT-1y/y) mice of OA models, the cartilage tissue changes are in line with previous reports. Moreover, bone defects (subchondral bone had less trabecular bone volume and thicker trabeculamicroM) and moderate local inflammations of the joint were also demonstrated in SIRT 1y/y mice [81]. In the SIRT 1−/− mice, MMP 13 and lymphoid enhancer-binding factor 1 (LEF1) appear to be elevated in the articular cartilage; activation of SIRT 1 plays a positive role in reducing the severity of OA, in part through its ability to repress the expression of MMPs [58]. Adult (9 month-old) heterozygous haploinsufficient SIRT 1 (+/−) mice showed decreased levels of aggrecan and other proteoglycans, but increased OA and levels of apoptosis compared with age-matched WT mice. Levels of full-length SIRT 1 were further decreased in both strains at 9 months. A 75 kDa SIRT 1 was found in 9-month-old WT mice; however, it was not detected in age-matched SIRT 1 (+/−) mice [83].

### Activation SIRT 1 inhibits the OA progress via resveratrol

Resveratrol, a SIRT 1 activator, can protect chondrocytes against OA development. Resveratrol increased SIRT 1 protein expression in a dose-dependent manner: at concentrations of 25 and/or 50 µM, resveratrol treatment significantly up-regulates SIRT 1 gene expression in normal and osteoarthritic chondrocytes [84]. This was blocked by the SIRT 1 inhibitor, sirtinol, which inhibits TNF-α-induced inflammatory factor COX-2 and MMPs release, as well as ECM degradation [46], Resveratrol protects the chondrocytes from IL-1β stimulation in a dose-dependent manner via its activation of SIRT 1 [85]. The inhibition of SIRT 1 enhances NO-induced apoptosis of human chondrocytes, and resveratrol inhibits this NO-induced apoptosis. Resveratrol reduced the amount of Bax and increased the amount of Bcl-2 in the mitochondrial fraction [24]. In rabbit with OA, intra-articular injection of melatonin significantly reduced cartilage degradation, which was reversed by sirtinol [64].

In human chondrocytes, the overexpression of SIRT1 plays a protective role through the NF-kB pathway, reducing the up-regulation of MMP 1, 2, 9, 13, and ADAMTS 5 genes caused by IL-1b [59]. Moreover, up-regulation of SIRT1 or treatment with the SIRT1 activator resveratrol could affect NF-kB expression caused by TNF-a in order to exert an anti-inflammatory effect on human chondrocytes [60]. Meanwhile, the elevation of SIRT1 positively affects cartilage genes including collagen 2a, collagen 2b, and aggrecan expression [38]. SIRT 1 up-regulation could also suppress OA chondrocyte apoptosis and ECM degradation through increasing Bcl-2 and decreasing Bax, MMP 1, and MMP 13 expression via the inhibition of p38, JNK, and ERK phosphorylation [86].

In experimental OA mice, treatment with the SIRT1 activator SRT1720 could attenuate OA development though inhibiting synovitis, partially inhibiting the declined COL2A1 and aggrecan, and decreasing MMP 13, ADAMTS 5, cleaved caspase 3, PARP p85, and acetylated NF-κB p65-positive chondrocytes [87]. Silencing miR-449a leads to the up-regulation of SIRT 1, promoting cartilage regeneration and preventing progression of OA in rat models [88].

In a double-blind, randomized control trial which included 110 people with mild-to-moderate knee OA in Iraq, the patient–subjects received 15 mg meloxicam and either 500 mg resveratrol or placebo per day for 90 days. The results showed that the pain severity and serum levels of biochemical markers were significantly decreased in the resveratrol-treated group compared with the placebo-treated group [89]. The study further showed that resveratrol significantly improved function and associated symptoms. 500 mg/day of resveratrol was safe and well-tolerated by the knee OA patients [90]. In France, a protocol for a multicenter randomized double-blind placebo-controlled trial to evaluate the knee OA patients’ pain after 3 months of taking oral resveratrol was published but the proceedings and the results have yet to be determined [91]. Consequently, the therapeutic effects of resveratrol or other SIRT 1 activators in practice require further investigation and validation in clinical trials.

## Conclusion

The greatest risk factor for OA is age. SIRT 1 is decreased with OA disease development in osteoarthritic cartilage. SIRT 1 can regulate ECM expression; promote MSCs differentiation; play anti-catabolic, anti-inflammatory, anti-oxidative stress, and anti-apoptosis roles; participate in the autophagic process; and regulate bone homeostasis in OA. Resveratrol activates SIRT 1 to inhibit the OA progress, in the future, activating SIRT 1 via resveratrol with better bioavailability may be an appropriate therapeutic approach for OA.

## Availability of data and materials

All data generated or analyzed during the present study are included in this published article.

## Abbreviations

ADAMTS: a disintegrin and metalloproteinase with thrombospondin motifs
AMPK: adenosine monophosphate-activated protein kinase
BM-MSCs: bone marrow-derived mesenchymal stem cells
COX-2: cyclooxygenase-2
DOT1L: disruptor of telomeric silencing 1-like
ECM: extracellular matrix
ERK: extracellular signal-regulated kinases
FOXOs: forkhead box transcription factor, class O
GlcN: glucosamine
HSF 1: heat shock factor 1
IGF: insulin-like growth factor
IGFR: insulin-like growth factor receptor
IL: interleukin
iNOS: inducible nitric oxide synthase
IR: ionizing radiation
JNK: C-Jun-N-terminal kinase
KO: knock-out
LEF1: lymphoid enhancer-binding factor 1
MAPK: mitogen-activated protein kinase
MDM2: mouse double minute 2 homolog
MMP: matrix metalloproteinase
MSC: mesenchymal stem cell
mTOR: mammalian target of rapamycin
NAD^+^: nicotinamide adenine dinucleotide
NAMPT: nicotinamide phosphoribosyltransferase
NF-κB: nuclear factor-k-gene binding
NO: nitric oxide
OA: osteoarthitis
p53: tumor protein p53
PDK1: pyruvate dehydrogenase kinase 1
per2: period 2
PGC-1: PPARγ coactivator-1
PGE2: prostaglandin E2
PHD: prolyl hydroxylase
PI3K: kinases phosphoinositide 3-kinases
PPARγ: peroxisome proliferator-activated receptor γ
PTHrP(1-84): parathyroid hormone-related protein 1–84
PTP1B: protein tyrosine phosphatase
ROS: reactive oxygen species
RUNX2: runt-related transcription factor 2
SIR 2: silent information regulator 2
SIRT: Sirtuin
SIRT 1: Sirtuin 1
TGF-β1: transforming growth factor-β1
TNF-α: tumor necrosis factor-α
SOX9: sex determining region Y box protein 9
Vav1: vav guanine nucleotide exchange factor 1
WT: wild-type
